# Supplementary material for: Association between Workers’ Anxiety over Technological Automation and Sleep Disturbance: Results from a Nationally Representative Survey
Source: Int J Environ Res Public Health. 2022 Aug 15;19(16):10051. doi: 10.3390/ijerph191610051 (PMC9408459; doi:10.3390/ijerph191610051)
Supplement: Supplementary file 1 [file ijerph-19-10051-s001.zip › ijerph-1848537-supplementary.pdf]

Supplementary Materials

Table S1. Scores for automation anxiety according to characteristics of the study samples. [SD=standard deviation].

Characteristics	N	Automation anxiety score (Mean ± SD, range:0-15)	P value[a]
Age groups (years)			
Young (≤ 35)	8,838	6.6 ± 3.6	< 0.001
Middle-aged (36-55)	20,641	6.6 ± 3.6	
Old (> 55)	17,046	6.0 ± 3.6	
Gender			
Men	21,833	6.5 ± 3.6	< 0.001
Women	24,692	6.3 ± 3.6	
Education			
Middle school or below	7,846	5.4 ± 3.4	< 0.001
High school	17,237	6.4 ± 3.6	
College or higher	21,442	6.7 ± 3.7	
Monthly income (1,000 ₩)			
≤ 2,000	15,652	5.7 ± 3.5	< 0.001
2,000-2,990	14,405	6.7 ± 3.6	
3,000-3,990	9,309	6.8 ± 3.6	
≥ 4,000	7,159	6.6 ± 3.7	
Occupation			
Blue collar	17,013	6.0 ± 3.6	< 0.001
Service/sales worker	14,004	6.6 ± 3.6	
White collar	15,508	6.6 ± 3.7	
Weekly working hours			
≤ 40	28,292	6.2 ± 3.6	< 0.001
41-52	10,732	6.7 ± 3.5	
> 52	7,501	6.8 ± 3.7	
Employment type			
Permanent	23,643	6.6 ± 3.6	< 0.001
Temporary/daily	7,115	5.8 ± 3.5	
Self-employed	14,395	6.5 ± 3.6	
Others	1,372	5.4 ± 3.5	
Shift work			
No	43,202	6.4 ± 3.6	0.177
Yes	3,323	6.3 ± 3.6	
Job stress			
Low	11,457	5.4 ± 3.6	< 0.001
Middle	21,450	6.6 ± 3.5	
High	13,618	7.0 ± 3.7	

Job satisfaction			
Low	7,989	6.5 ± 3.6	0.049
High	38,536	6.4 ± 3.6	
Facing with angry customers			
Rarely	39,290	6.3 ± 3.7	< 0.001
Sometimes	5,580	6.9 ± 3.4	
Always	1,655	6.6 ± 3.4	

[a]ANOVA or Student's t-test.

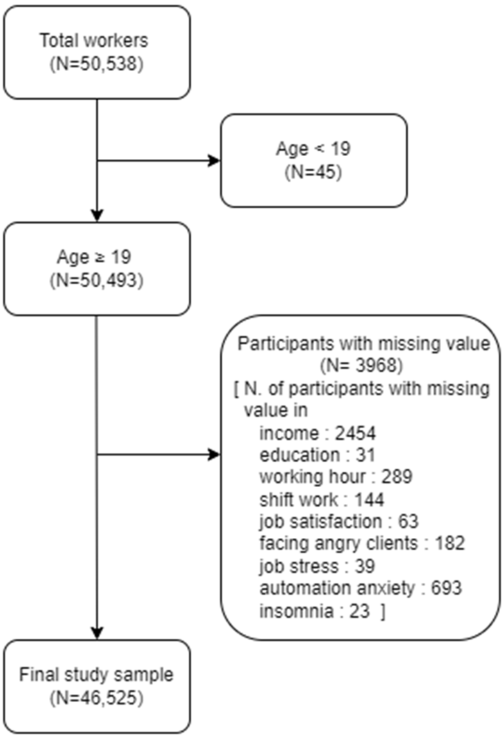

Figure S1. Flowchart of selection of study sample
